# Galloyl‐bis‐hexahydroxydiphenoyl glucose and endothelial remodelling: nutrigenomic insights into blackberry's superior vasorelaxant efficacy over mugwort

**DOI:** 10.1113/EP092901

**Published:** 2025-06-18

**Authors:** Shan Tao, Yinuo Qin, Chengqiang Jin

**Affiliations:** ^1^ School of Medical Imaging and Laboratory Medicine Jining Medical University Jining Shandong Province China; ^2^ Medical Laboratory Department Affiliated Hospital of Jining Medical University Jining Shandong Province China

**Keywords:** *Artemisia campestris*, endothelial remodelling, galloyl‐bis‐HHDP glucose, nutrigenomics, *Rubus ulmifolius*, vasorelaxant efficacy

1

The study by Mehiou et al. (2025) elegantly integrates phytochemical, physiological and transcriptomic approaches to validate the antihypertensive properties of *Rubus ulmifolius* (blackberry) and *Artemisia campestris* (mugwort). By identifying galloyl‐bis‐hexahydroxydiphenoyl (HHDP) glucose derivatives as a unique vasoactive compound in blackberry and linking Tfcp2l1 upregulation to endothelial maturation, the authors provide unprecedented mechanistic insights into traditional herbal therapies. Their work exemplifies the synergy between ethnopharmacology and modern omics technologies, solidifying *Experimental Physiology*’s reputation as a beacon of translational research.

This multidisciplinary study stands as a paradigm for ethnopharmacological research, masterfully combining dose‐dependent in vivo hypotensive assays, *ex vivo* vascular reactivity experiments, and hepatic transcriptomic profiling. The authors’ discovery of blackberry's 20‐fold greater vasorelaxant efficacy compared to mugwort, attributed to its unique phytochemical composition and endothelial gene modulation (*Amotl2*, *Cdh1*), underscores the rigor and novelty of their approach. Such comprehensive validation of traditional remedies through contemporary molecular frameworks not only honours ancestral knowledge but also sets a benchmark for future studies in nutrigenomics.

While the study is exemplary, two limitations warrant attention for translational refinement.

The nutrigenomic analysis centred on hepatic tissue, whereas vascular endothelial cells are the direct mediators of the observed vasodilatory effects. A targeted transcriptomic approach, such as single‐cell RNA sequencing of aortic endothelium, could elucidate localized gene networks driving vascular remodelling. For instance, Kalucka et al. (2020) employed this technique to map endothelial heterogeneity in murine tissues, revealing distinct metabolic and functional signatures across vascular beds. Their work demonstrated how endothelial subpopulations differentially regulate nitric oxide synthesis and vascular tone in response to stimuli. Adopting similar methodologies would clarify whether *Tfcp2l1* or *Amotl2* upregulation directly enhances endothelial function in a vessel‐specific manner, bridging the gap between hepatic gene expression and site‐specific vascular outcomes.

Although the phytochemical profile identified key bioactive compounds (e.g., galloyl‐bis‐HHDP glucose), the absence of pharmacokinetic data limits mechanistic clarity. Quantitative tracking of compound bioavailability and tissue distribution, as demonstrated by Shang et al. (2018) using liquid chromatography–tandem mass spectrometry to monitor flavonoid metabolites in plasma, would strengthen claims about bioactivity. Such data could also inform optimal dosing regimens for preclinical trials, ensuring therapeutic efficacy without toxicity.

Mehiou et al. have delivered a landmark study that harmonizes traditional wisdom with 21st‐century science. Addressing the above gaps through endothelial‐specific omics and pharmacokinetic profiling will further elevate its impact, paving the way for clinical applications. This work not only enriches our understanding of herbal therapeutics but also reaffirms *Experimental Physiology*’s pivotal role in advancing integrative physiological research.

## AUTHOR CONTRIBUTIONS

Conceptualization, Writing—Original Draft: Shan Tao and Yinuo Qin. Validation, Writing–Review & Editing: Chengqiang Jin. All authors have read and approved the final version of this manuscript and agree to be accountable for all aspects of the work in ensuring that questions related to the accuracy or integrity of any part of the work are appropriately investigated and resolved. All persons designated as authors qualify for authorship, and all those who qualify for authorship are listed.

## CONFLICT OF INTEREST

No potential conflicts of interest relevant to this article were reported.

## FUNDING INFORMATION

This work is supported by the Undergraduate Innovation Training Program of Jining Medical University (Grant No. 202206040101). Practice Teaching and Education Research Program of Jining Medical University (JYSJ2024C35).
